# Supplementary material for: Role and mechanism of NCAPD3 in promoting malignant behaviors in gastric cancer
Source: Front Pharmacol. 2024 Apr 22;15:1341039. doi: 10.3389/fphar.2024.1341039 (PMC11070777; doi:10.3389/fphar.2024.1341039)
Supplement: Supplementary file 11 [file DataSheet2.ZIP › GSEA/GO_CC/my_analysis.Gsea.1599461408869/GO_SPECIFIC_GRANULE.html]

Details for gene set GO\_SPECIFIC\_GRANULE[GSEA]

|  || Dataset | filtered\_dataset.sample\_info.cls#WT\_versus\_NCAPD3\_MUT |
| Phenotype | sample\_info.cls#WT\_versus\_NCAPD3\_MUT |
| Upregulated in class | NCAPD3\_MUT |
| GeneSet | GO\_SPECIFIC\_GRANULE |
| Enrichment Score (ES) | -0.40587932 |
| Normalized Enrichment Score (NES) | -1.7177576 |
| Nominal p-value | 0.029268293 |
| FDR q-value | 0.1088227 |
| FWER p-Value | 0.514 |
Table: GSEA Results Summary

  

Fig 1: Enrichment plot: GO\_SPECIFIC\_GRANULE      
 Profile of the Running ES Score & Positions of GeneSet Members on the Rank Ordered List

  

| SYMBOL | TITLE | RANK IN GENE LIST | RANK METRIC SCORE | RUNNING ES | CORE ENRICHMENT || 1 | 81619 | TSPAN14 | 212 | 0.666 | -0.0798 | No |
| 2 | 25852 | ARMC8 | 307 | 0.588 | -0.0835 | No |
| 3 | 2919 | CXCL1 | 405 | 0.534 | -0.0951 | No |
| 4 | 23250 | ATP11A | 664 | 0.395 | -0.2372 | No |
| 5 | 6515 | SLC2A3 | 873 | -0.283 | -0.3557 | No |
| 6 | 4973 | OLR1 | 944 | -0.366 | -0.3663 | Yes |
| 7 | 5329 | PLAUR | 952 | -0.373 | -0.3309 | Yes |
| 8 | 966 | CD59 | 1040 | -0.436 | -0.3460 | Yes |
| 9 | 6590 | SLPI | 1062 | -0.453 | -0.3120 | Yes |
| 10 | 6709 | SPTAN1 | 1114 | -0.487 | -0.2957 | Yes |
| 11 | 6947 | TCN1 | 1135 | -0.503 | -0.2556 | Yes |
| 12 | 5795 | PTPRJ | 1210 | -0.586 | -0.2451 | Yes |
| 13 | 7414 | VCL | 1308 | -0.712 | -0.2375 | Yes |
| 14 | 1116 | CHI3L1 | 1339 | -0.777 | -0.1749 | Yes |
| 15 | 2040 | STOM | 1389 | -0.988 | -0.1029 | Yes |
| 16 | 2444 | FRK | 1396 | -1.082 | 0.0100 | Yes |
Table: GSEA details [plain text format]

  

Fig 2: GO\_SPECIFIC\_GRANULE      
 Blue-Pink O' Gram in the Space of the Analyzed GeneSet

  

Fig 3: GO\_SPECIFIC\_GRANULE: Random ES distribution      
 Gene set null distribution of ES for **GO\_SPECIFIC\_GRANULE**
